# The impact of nicotine on the olfactory memory and its relationship with TRPA1

**DOI:** 10.1016/j.isci.2026.114908

**Published:** 2026-02-07

**Authors:** Kazuya Mizobata, Hideki Sakatani, Masamitsu Kono, Shizuya Saika, Akinori Akaike, Muneki Hotomi

**Affiliations:** 1 Department of Otorhinolaryngology-Head and Neck Surgery, Wakayama Medical University, Kimiidera 811-1, Wakayama 641-8509, Japan; 2 Department of Ophthalmology, Wakayama Medical University, 811-1 Kimiidera, Wakayama 641-8509, Japan; 3 Department of Pharmacology, Graduate School of Pharmaceutical Sciences, Wakayama Medical University, 811-1 Kimiidera, Wakayama 641-8509, Japan

**Keywords:** Biological sciences, Neuroscience, Cell biology

## Abstract

Olfactory disorders are related to cognitive decline, yet effective treatments remain limited. Cigarette smoke impairs olfaction, but one of its components, nicotine, can protect nerve cells in the central nervous system. Transient receptor potential ankyrin 1 (TRPA1) is expressed in central olfactory tract conduction and can be activated by nicotine. We therefore investigated whether nicotine influences olfaction through TRPA1. After inducing olfactory damage with methimazole, one of the following agents was then administered for 42 days to wild-type (WT) and TRPA1 knockout (TRPA1 KO) mice: phosphate-buffered saline, cigarette smoke extract, or nicotine. Olfactory behavioral testing and histological analyses were performed. In WT mice, intraoral nicotine improved olfactory memory and reduced neuronal apoptosis in the piriform cortex (PC). In TRPA1 KO mice, however, these effects of nicotine were abolished. Nicotine improved olfaction through TRPA1 in central olfactory tract conduction. Nicotine may thus represent a potential therapeutic strategy for olfactory impairment.

## Highlights

• Oral nicotine treatment improves olfactory memory in mice
• Nicotine reduces neuronal apoptosis in the piriform cortex
• TRPA1 knockout abolishes nicotine’s beneficial effect
• Nicotine’s neuroprotective action in the piriform cortex is TRPA1-dependent

## Graphical abstract

**Figure:**
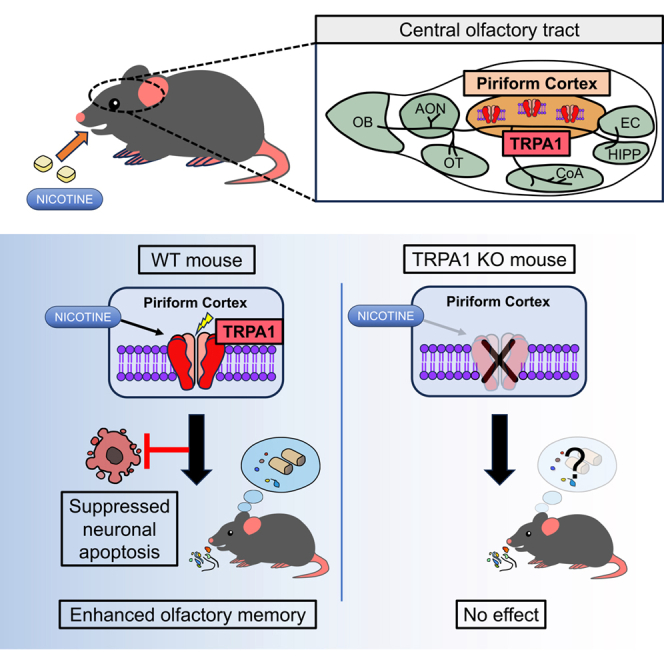

## Introduction

Olfactory disorders are especially common in elderly people.^1^ They are a precursor to several neurodegenerative diseases that impair cognitive function, including Alzheimer’s disease.^2^^,^^3^^,^^4^^,^^5^^,^^6^^,^^7^ Furthermore, they can lead to sarcopenia through depression and loss of appetite, which in turn exacerbates cognitive impairment.^8^ In countries with aging populations, olfactory disorders represent an important medical issue that is closely linked to dementia. However, effective treatments are currently lacking for neuronal olfactory disorders, including those that are related to age.

The nasal cavity contains a specialized mucosa called the olfactory epithelium (OE), which houses olfactory receptor neurons (ORNs).^9^^,^^10^ Odorants bind to olfactory receptors located on the cilia of the OE.^11^ Odor signals are conveyed via the axons to the olfactory bulb (OB) and are subsequently transmitted to higher cortical regions.^12^ The olfactory higher cortex comprises the anterior olfactory nucleus (AON), olfactory tubercle, piriform cortex (PC), cortical amygdala (CoA), and entorhinal cortex. Among them, the PC has the largest volume and is involved in olfactory memory.^13^^,^^14^^,^^15^ Through this olfactory pathway, additional information is added, such as emotions and memories.^16^^,^^17^ Neural olfactory disorders are classified as “sensorineural disorders,” which involve dysfunction of the ORNs, or “central disorders,” which involve impairment within the central nervous system.^18^^,^^19^ Accordingly, the olfactory tract conduction can be categorized into “peripheral,” which involves the ORNs, or “central,” which involves the olfactory higher cortex.

Cigarette smoke is generally considered to be harmful, although habitual smoking may actually exert neuroprotective effects. This notion is based on the theory that nicotine in cigarettes chronically stimulates nicotinic acetylcholine receptors in the brain.^20^ Acetylcholinergic neurons are present in the central olfactory tract conduction.^21^^,^^22^ Cigarette smoke has been indicated in several reports to impair olfaction,^23^^,^^24^^,^^25^ but nicotine alone has been reported to improve olfactory memory of honeybees,^26^ and olfaction of Alzheimer’s disease model mice.^27^ In a clinical trial, nicotine enhanced blood flow in the central olfactory tract conduction.^28^ By excluding non-nicotine toxicants present in cigarettes, nicotine itself may improve olfactory function. E-cigarettes have recently gained in popularity in place of conventional heated tobacco. Although containing fewer harmful substances (such as tar) than conventionally heated tobacco, e-cigarettes still contain nicotine, but their effects on health are still unclear.

Transient receptor potential ankyrin 1 (TRPA1), a member of the TRP superfamily, is a polymodal and non-selective cation channel. It is widely expressed in both neuronal and non-neuronal cell types,^29^^,^^30^ but its expression is highest in the central nervous system.^31^ TRPA1 functions as a detector of various changes, including temperature, mechanical stimuli, and hypoxia.^32^^,^^33^ Interestingly, nicotine is a known TRPA1 agonist.^34^ As TRPA1 is expressed within the central olfactory tract conduction and is involved in the central processing of olfaction,^35^ we hypothesized that investigating the effects of nicotine on olfactory tract conduction via TRPA1 could lead to novel therapeutic strategies for olfactory disorders.

In this study, we used a methimazole-induced OE damage mouse model to investigate the effects of cigarette smoke and nicotine on olfaction, as well as the involvement of TRPA1. Our aim was to provide new insights into how nicotine and cigarette smoke influence olfaction, with the goal of developing novel therapeutic strategies for olfactory disorders.

## Results

### The effects of CSE and nicotine on peripheral olfactory tract conduction in WT mice

**Figure 1:**
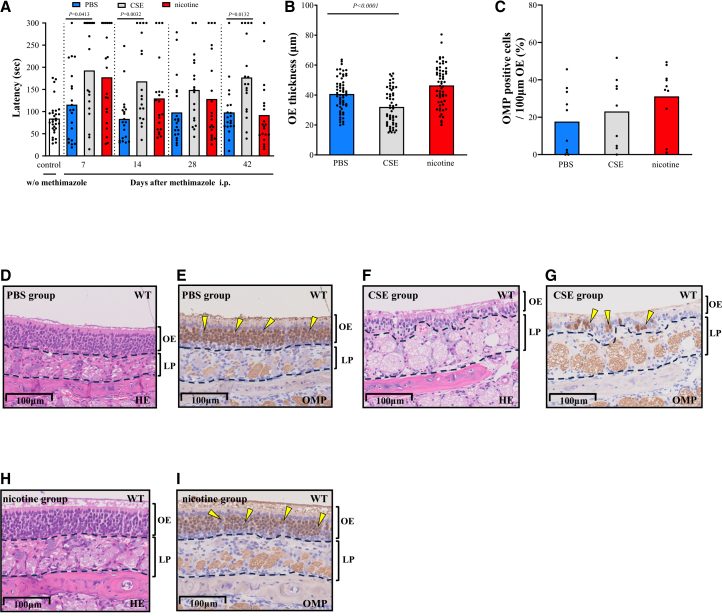
The effects of CSE and nicotine on peripheral olfactory tract conduction in WT mice (A) Time course changes in buried food test after methimazole administration. The shorter latency meant better olfactory detection. Buried food test was carried out on two consecutive days, with two dots plotted per mouse on each test day. Control mice before methimazole administration (*n* = 15), PBS group (*n* = 10), CSE group (*n* = 9), and nicotine group (*n* = 10). (B) OE thickness on day 42. Six dots per mouse are plotted. PBS group (*n* = 10), CSE group (*n* = 9), and nicotine group (*n* = 10). (C) OMP-positive cell rate on day 42. PBS group (*n* = 10), CSE group (*n* = 9), and nicotine group (*n* = 10). Bars represent the mean. Statistical comparisons to PBS group were performed using Kruskal-Wallis test with Dunn’s multiple-comparison test. (D–I) Representative histological images of OE on day 42. (D and E) PBS group. (F and G) CSE group. (H and I) nicotine group. (D, F, and H) HE-stained images. (E, G, and I) anti-OMP antibody-stained images. The yellow arrowheads represent ORN cell bodies. The scale bars at the bottom left represent 100 μm. CSE, cigarette smoke extract; PBS, phosphate-buffered saline; LP, lamina propria; OE, olfactory epithelium; OMP, olfactory marker protein, HE, hematoxylin-eosin.

To investigate the effects of cigarettes and nicotine on olfactory tract conduction, we first focused on ORNs, which constitute the peripheral olfactory tract conduction. Buried food tests were performed using wild-type (WT) mice prior to methimazole administration as controls. The latency was significantly longer in the cigarette smoke extract (CSE) group than in the phosphate-buffered saline (PBS) group on days 7, 14, and 42. There were no significant differences between the nicotine and PBS group (Figure 1A). Regarding OE thickness on day 42, the CSE group exhibited predominantly thinner OE, while the nicotine group showed no significant difference compared with the PBS group (Figure 1B). Olfactory marker protein (OMP) is an antigen specific to ORNs, and OMP-positive cells represent ORNs within OE.^36^^,^^37^ The proportion of OMP-positive cells within the OE did not differ among the groups (Figures 1C–1I). CSE exacerbated olfactory detection and OE damage, while nicotine itself had no effect.

### Oral nicotine administration improved function of central olfactory tract conduction in WT mice

**Figure 2:**
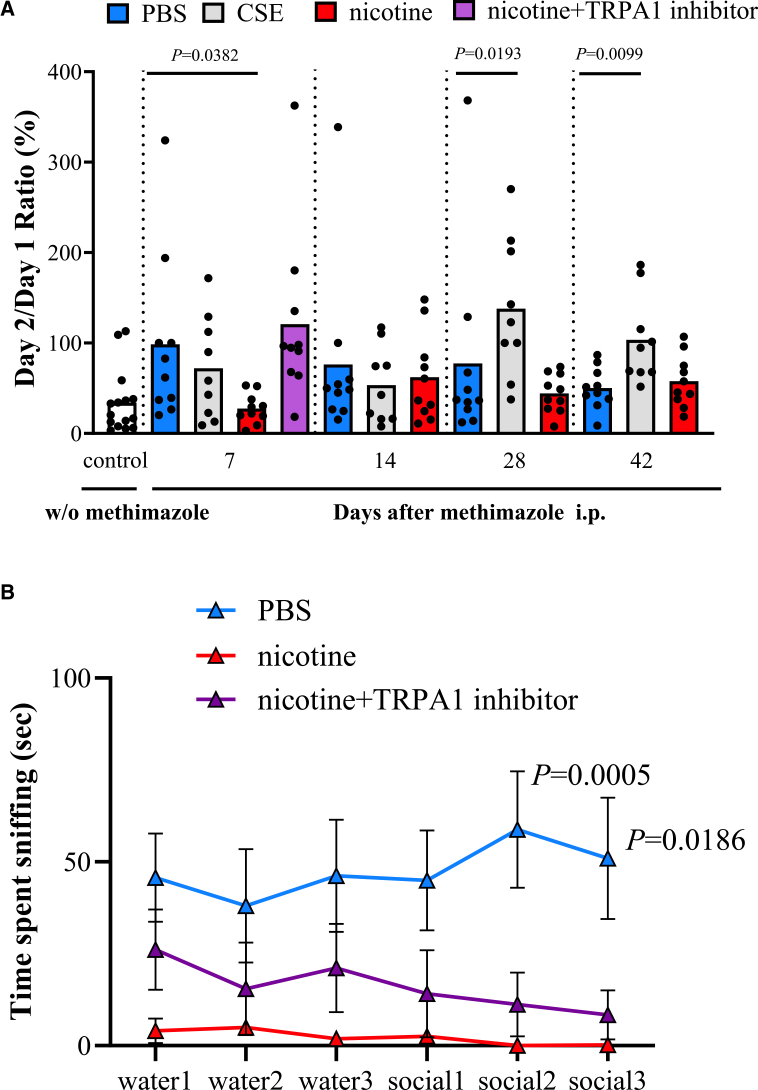
The effects of CSE and nicotine on central olfactory tract conduction in WT mice (A) Olfactory memory test. The ratio of two consecutive days, per mouse. A small day 2/day 1 ratio indicates good olfactory memory. One dot represents one mouse. Control mice before methimazole administration (*n* = 15), PBS group (*n* = 10), CSE group (*n* = 9), nicotine group (*n* = 10), and the nicotine + TRPA1 inhibitor subgroup (*n* = 10). Bars represent the mean. (B) Olfactory habituation/dishabituation on day 7. The triangles represent average time spent sniffing of PBS group (*n* = 9), nicotine group (*n* = 8), and the nicotine + TRPA1 inhibitor subgroup (*n* = 9). Error bars indicate the standard error of the mean. Oral nicotine administration improved function of central olfactory tract conduction in WT mice. Statistical comparisons to PBS group were performed using Kruskal-Wallis test with Dunn’s multiple-comparison test (A) and multiple Mann-Whitney U tests (B). CSE, cigarette smoke extract; PBS, phosphate-buffered saline.

Next, to investigate the effects on central olfactory tract conduction, we performed olfactory memory test and olfactory habituation/dishabituation test in WT mice. The nicotine group showed significantly lower day 2/day 1 ratio than the PBS group on day 7, indicating that olfactory memory was improved in the nicotine group. In the nicotine + TRPA1 inhibitor subgroup, this improvement was no longer observed compared with the PBS group. In contrast, the CSE group showed a significantly higher day 2/day 1 ratio on days 28 and 42 compared with the PBS group (Figure 2A). In habituation/dishabituation tests, the nicotine group spent less time investigating social odor than the PBS group on day 7, indicating a significant habituation response.^38^ No significant difference from the PBS group was observed in the nicotine + TRPA1 inhibitor subgroup (Figure 2B). Anxiety is a potential confounding factor in olfactory behavioral tests, so we assessed anxiety levels using the avoidance behavioral test.^39^ On day 7, there were no significant differences in anxiety levels among the PBS, CSE, and nicotine groups (Figure S1). Furthermore, to assess the effect of systemic nicotine administration, we compared the nicotine intraperitoneal (i.p.) group with the PBS i.p. group, and there were no significant differences in olfactory memory, OE thickness, or OMP-positive cell ratio (Figures S2A–S2C). Oral nicotine administration was indicated by these results to improve the function of central olfactory tract conduction in WT mice.

### Apoptosis in central olfactory tract conduction was inhibited by oral nicotine treatment

**Figure 3:**
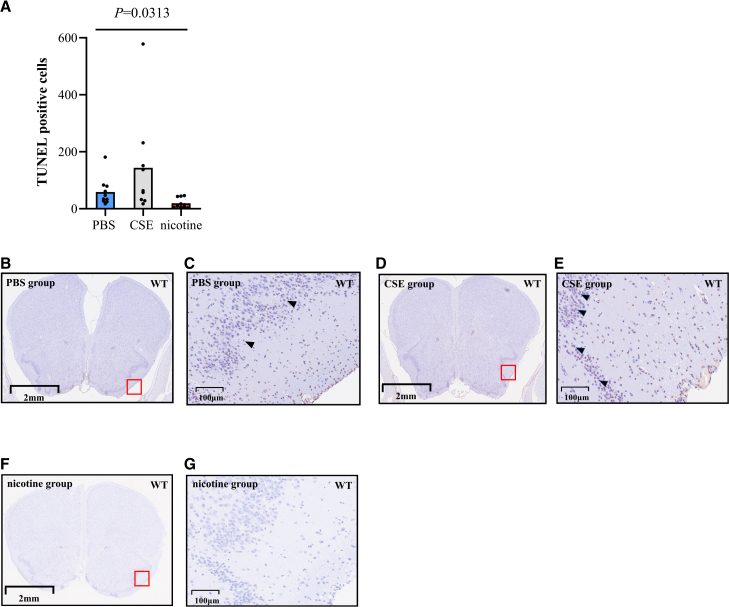
Apoptosis in central olfactory tract conduction was inhibited by oral nicotine treatment (A) Number of TUNEL stain-positive cells. PBS group (*n* = 10), CSE group (*n* = 9), and nicotine group (*n* = 10). Data are presented as individual dots and bars represent the mean. Statistical comparisons to PBS group were performed using Kruskal-Wallis test with Dunn’s multiple-comparison test. (B–G) Representative coronal images of the brain, including the PC. (B and C) PBS group. (D and E) CSE group. (F and G) nicotine group. (B, D, and F) Low-magnification images. The areas enclosed by red lines indicate representative regions of the PC. Scale bars at the bottom left represent 2 mm. (C, E, and G) Highly magnified images of the red boxes in (B), (C), and (D). Black arrowheads represent TUNEL stain-positive cells in PC. Scale bars at the bottom left represent 100 μm. Apoptosis of neurons in PC was histologically analyzed using TUNEL staining on day 42 in WT mice. PC, piriform cortex; CSE, cigarette smoke extract; PBS, phosphate-buffered saline.

Next, we investigated how nicotine and CSE affected the central olfactory tract conduction. We examined apoptosis within the central olfactory tract conduction using coronal brain sections including the PC on day 42. The nicotine group showed a significantly lower number of TUNEL stain-positive cells than the PBS group. The number of TUNEL stain-positive cells in the CSE group did not differ from that in the PBS group (Figure 3A). In representative histological brain images, numerous TUNEL-positive cells (black arrowheads) were observed around the PC in the PBS and CSE groups (Figures 3B–3E), whereas comparatively few were observed in the nicotine group (Figures 3F and 3G).

### TRPA1 was present in the OE, OB, and PC

**Figure 4:**
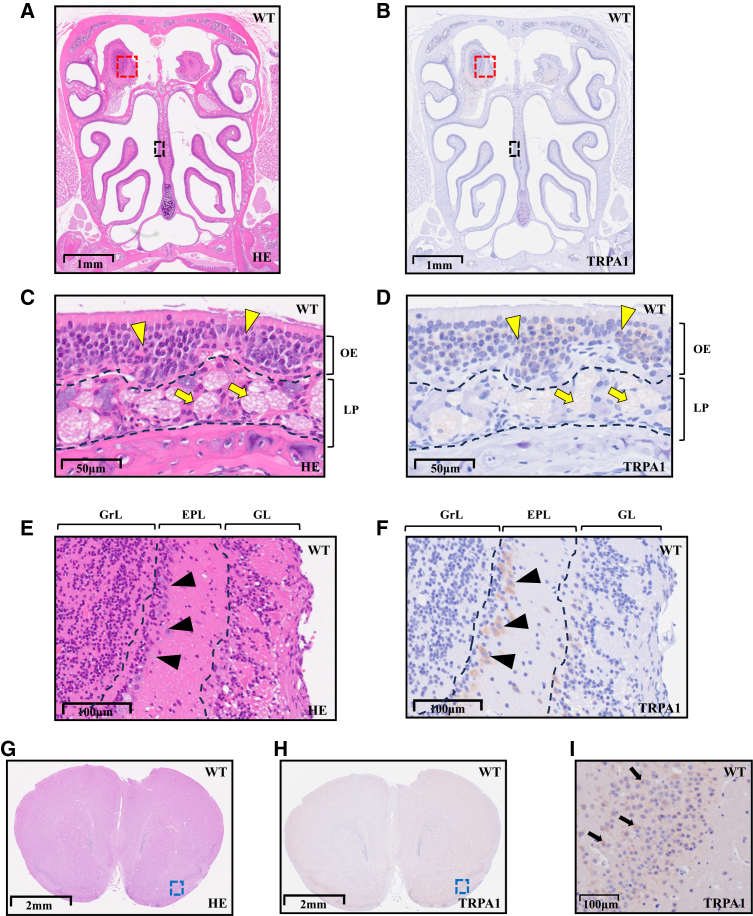
Representative histological images of WT control mice before methimazole administration (A–F) Coronal sections of nasal cavity. (G–I) Coronal sections of brain including the PC. TRPA1 was present in the olfactory epithelium, olfactory bulb, and piriform cortex. (A, C, E, and G) HE-stained images. (B, D, F, H, and I) Anti-TRPA1 antibody-stained images. (A and B) Low-magnification images of nasal cavity. The area surrounded by the black and red dashed lines represents the OE and the OB, respectively. (C and D) Highly magnified images of OE in the black boxes in (A and B). Yellow arrowheads and yellow arrows indicate ORN cell bodies and olfactory nerve fascicles, respectively. (E and F) Highly magnified images of the OB in the red boxes of (A and B). Black arrowheads represent TRPA1-positive cells in the EPL. (G and H) Low-magnification images of brain. The area enclosed by the blue dashed line represents one part of the PC. (I) Highly magnified image of PC in the blue box of H. The black arrows represent the cell bodies of the PC. Scale bars represent (A and B) 1 mm, (C and D) 50 μm, (E and F) 100 μm, (G and H) 2 mm, and (I) 100 μm. TRPA1, transient receptor potential ankyrin 1; HE, hematoxylin-eosin; OE, olfactory epithelium; OB, olfactory bulb; LP, lamina propria; GrL, granule cell layer; EPL, external plexiform layer; GL, glomerular layer; PC, piriform cortex.

We hypothesized that the effect of nicotine on olfaction would be mediated by TRPA1. We therefore histologically investigated the localization of TRPA1 in the olfactory tract conduction. In WT control mice before methimazole administration, TRPA1 was expressed in the OE and in the OB (Figures 4A and 4B). Magnified images showed faint TRPA1 expression in ORN cell bodies (yellow arrowheads) and in olfactory nerve fascicles (yellow arrows) (Figures 4C and 4D), as well as moderate expression in cells of the external plexiform layer in the OB (black arrowhead) (Figures 4E and 4F). In the olfactory higher cortex, TRPA1 was detected in the cell bodies (black arrows) in the PC (blue dashed line area) (Figures 4G–4I). These TRPA1 immunoreactivities were largely absent in TRPA1 knockout (KO) control mice before methimazole administration (Figure S3).

### In TRPA1 KO mice, the effects of CSE and nicotine on peripheral olfactory tract conduction were similar to those in WT mice

**Figure 5:**
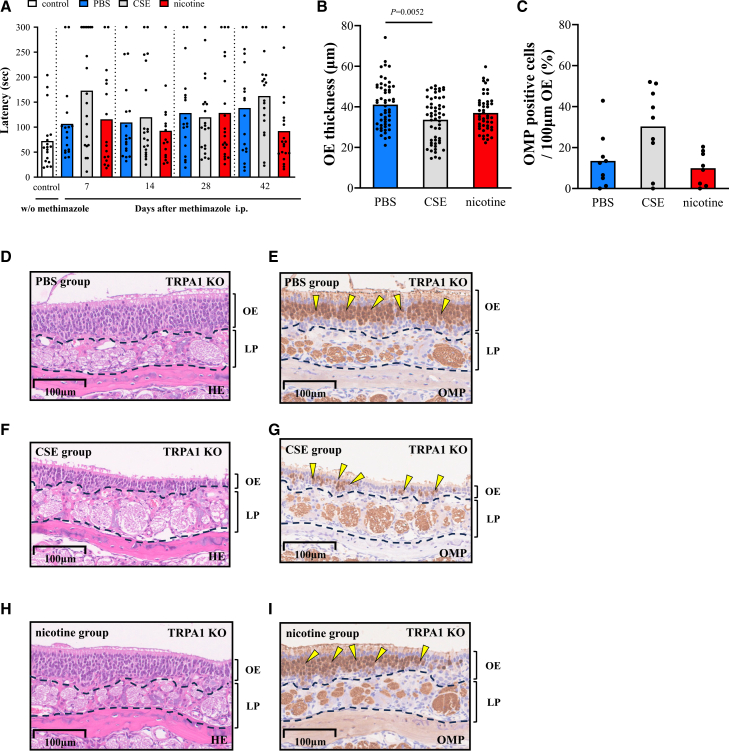
The effects of CSE and nicotine on peripheral olfactory tract conduction in TRPA1 KO mice (A) Time course changes in buried food test after methimazole administration. Two dots plotted per mouse on each test day. Control mice before methimazole administration (*n* = 9), PBS group (*n* = 9), CSE group (*n* = 10), and nicotine group (*n* = 8). (B) OE thickness on day 42. Six dots per mouse are plotted. PBS group (*n* = 9), CSE group (*n* = 9), and nicotine group (*n* = 8). (C) OMP-positive cell rate on day 42. PBS group (*n* = 9), CSE group (*n* = 9), and nicotine group (*n* = 8). Bars represent the mean. Statistical comparisons to PBS group were performed using Kruskal-Wallis test with Dunn’s multiple-comparison test. (D–I) Representative histological images of OE on day 42. (D and E) PBS group. (F and G) CSE group. (H and I) nicotine group. (D, F, and H) HE-stained images. (E, G, and I) anti-OMP antibody-stained images. Yellow arrowheads represent ORN cell bodies. The scale bars at the bottom left represent 100 μm. In TRPA1 KO mice, the effects of cigarette smoke extract and nicotine on peripheral olfactory tract conduction were similar to those in WT mice. CSE, cigarette smoke extract; PBS, phosphate-buffered saline; LP, lamina propria; OE, olfactory epithelium; OMP, olfactory marker protein, HE, hematoxylin-eosin.

To determine whether the effects of nicotine were mediated by TRPA1, buried food tests and histological analyses of the OE were performed in TRPA1 KO mice and in WT mice. Similar to the WT mice in Figure 2A, buried food tests in TRPA1 KO mice showed no significant differences between the PBS and nicotine groups during the observation period (Figure 5A). OE thickness was greater in the PBS group than in the CSE group, and was comparable to that of the nicotine group on day 42 (Figure 5B); these trends were similar to those shown in Figure 1B. There were no significant differences in the proportion of OMP-positive cells among the groups (Figures 5C–5I). The effects of nicotine and CSE on peripheral olfactory tract conduction were indicated by these results to be largely independent of TRPA1.

### TRPA1 KO abolished the nicotine-induced improvement in central olfactory tract conduction

**Figure 6:**
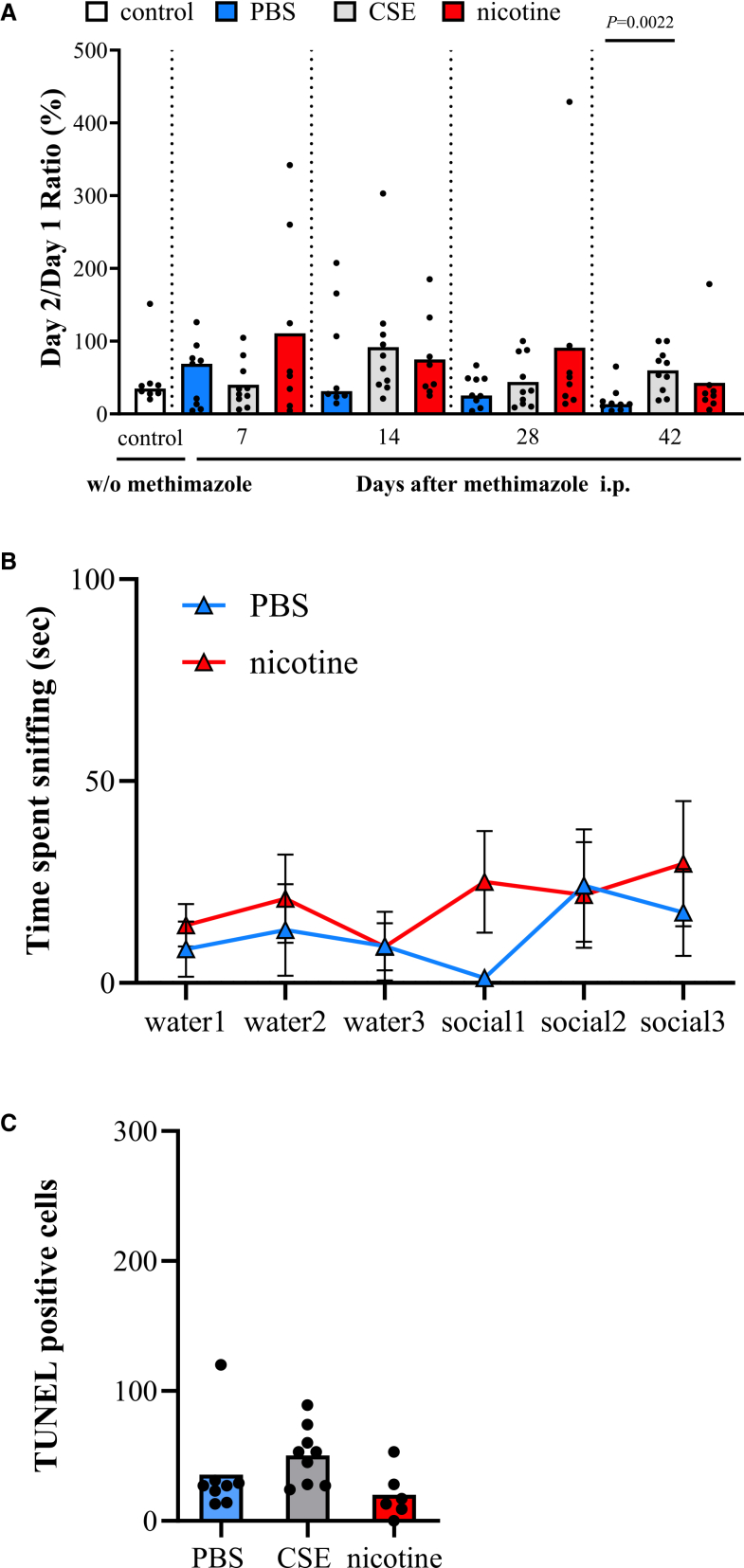
The effects of CSE and nicotine on central olfactory tract conduction in TRPA1 KO mice (A) Olfactory memory test. The ratio of two consecutive days per mouse. One dot represents one mouse. Control mice before methimazole administration (*n* = 8), PBS group (*n* = 9), CSE group (*n* = 10), and nicotine group (*n* = 8). Bars represent the mean. (B) Olfactory habituation/dishabituation on day 7. The triangles represent average time spent sniffing of PBS group (*n* = 9) and nicotine group (*n* = 9). Error bars indicate the standard error of the mean. (C) Number of TUNEL stain-positive cells of TRPA1 KO mice on day 42. PBS group (*n* = 8), CSE group (*n* = 9), and nicotine group (*n* = 6). Data are presented as individual dots and bars represent the mean. TRPA1 KO abolished the nicotine-induced improvement in central olfactory tract conduction. Statistical comparisons to PBS group were performed using Kruskal-Wallis test with Dunn’s multiple-comparison test in (A) and (C), and multiple Mann-Whitney U tests in (B).

TRPA1 KO mice were subjected to the same central olfactory tract conduction assessments as WT mice. Olfactory memory tests showed no significant differences between the nicotine and PBS groups throughout the observation period (Figure 6A). The nicotine-induced improvement observed in WT mice (Figure 2A) was indicated by this finding to be absent in TRPA1 KO mice. On day 42, the CSE group showed significantly poorer olfactory memory than the PBS group (Figure 6A), which is consistent with findings in WT mice (Figure 2A). In habituation/dishabituation tests, TRPA1 KO mice showed comparable responses to social stimuli in the PBS and nicotine groups (Figure 6B), unlike the WT mice (Figure 2B). The number of TUNEL stain-positive cells on day 42 was similar between the nicotine and PBS groups (Figure 6C), unlike the difference observed in WT mice (Figure 3A). TRPA1 KO was indicated by these results to have abolished the effects of nicotine on central olfactory tract conduction. To rigorously assess the interaction between genotype and treatment, two-way ANOVA would ideally be performed on selected tests in which nicotine treatment effects were different between WT mice and TRPA1 KO mice. However, even after applying data transformations such as logarithmic transformation, the datasets did not meet the assumptions of normality or homogeneity of variance. Therefore, the statistical analysis was restricted to non-parametric tests.

## Discussion

We examined the effects of cigarette smoke and nicotine on peripheral and central olfactory tract conduction. Methimazole specifically impairs ORNs (peripheral tract conduction) and secondarily affects central tract conduction.^40^^,^^41^^,^^42^ Methimazole-induced olfactory impairment is therefore considered a suitable model of neural olfactory disorders, such as those caused by aging or viral infections. The buried food test in this study showed that cigarette smoke exacerbated peripheral olfactory tract conduction under neural olfactory disorder. Cigarette smoke has been shown to impair ORNs in aged mice, contributing to age-related olfactory dysfunction.^43^ Our findings indicate that cigarette smoke influenced neural olfactory disorders in general, and it was not limited to age-related olfactory dysfunction. A nicotine dose of 2 μg/g in mice corresponds to 0.16 mg/kg in humans, which is equivalent to about 13 mg for an 80 kg adult.^44^ For comparison, nicotine gum for nicotine replacement therapy is typically administered at 16–48 mg per day for 6–12 weeks.^45^ In this study, both the dose and duration of treatment were set below the lower limit of the standard clinical regimen to minimize potential side effects. Various formulations of nicotine are clinically available, so both oral and intraperitoneal administration routes were tested in this study for exploratory purposes. In humans, the blood half-life of intravenously injected nicotine is approximately 20 min,^46^ whereas that of orally-administered nicotine is approximately 2–3 h.^47^ A longer blood half-life is thus suggested to be required for nicotine to exert effects on olfactory pathways in mice. Our findings suggest that oral nicotine formulations used in nicotine replacement therapy may also be applicable for improving olfactory memory.

Olfactory memory comprises both long-term and short-term components, and involves multiple brain regions, including the OB, PC, entorhinal cortex, and hippocampus.^48^ Olfactory memory tests primarily reflect short-term memory.^12^ Olfactory memory was poorer in the PBS group on day 7 than in control mice (*p* = 0.012, Mann-Whitney U test) (Figure 2A), which indicates that methimazole affected central tract conduction secondarily to peripheral tract conduction. On day 7, the nicotine group showed better olfactory memory (Figure 2A), despite exhibiting no differences in peripheral olfactory tract function compared with the PBS group (Figures 1A–1C). Oral nicotine treatment is suggested by these results to have primarily affected central olfactory pathways. The disappearance of significant differences after day 7 may be attributable to functional recovery due to ORN regeneration over time, as well as to adaptation to the test.

Cigarettes contain various toxic substances.^49^^,^^50^ Tar, for example, generates free radicals that can damage the cerebral cortex through oxidative stress,^51^ and it induces apoptosis and inhibits regeneration of the OE.^43^ The poor olfactory memory observed in the CSE group was likely to be due to a combination of central olfactory pathway dysfunction and ORN impairment. The beneficial effects of nicotine were probably outweighed by the presence of other toxic components.

**Figure 7:**
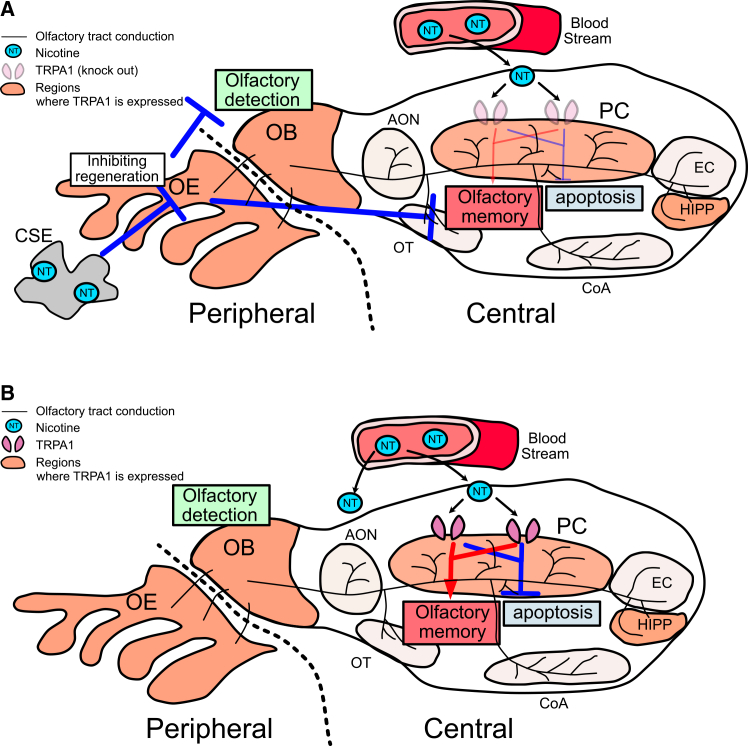
Schematic diagram of the effects of cigarette smoke extract and nicotine on olfactory tract conduction (A) In TRPA1 KO mice, CSE damages peripheral olfactory tract conduction by inhibiting OE regeneration. Nicotine in CSE can show little inhibitory effect on apoptosis in PC under TRPA1 KO and cannot improve olfactory memory. The impairment of central olfactory tract conduction due to CSE therefore outweighs the effects of nicotine. (B) WT mice. Nicotine administered alone to WT mice has no direct effect on peripheral olfactory tract conduction. In contrast, via the bloodstream, nicotine acts on TRPA1 in PC to inhibit apoptosis and improves olfactory memory. Olfactory tract conduction is divided into “peripheral,” involving OE and ORNs, and “central,” involving the central nervous system. TRPA1, transient receptor potential ankyrin 1; OE, olfactory epithelium; OB, olfactory bulb; CSE, cigarette smoke extract; NT, nicotine; AON, anterior olfactory nucleus; OT, olfactory tubercle; PC, piriform cortex; CoA, cortical amygdala; EC, entorhinal cortex; HIPP, hippocampus.

We sought to clarify why oral nicotine treatment improved olfactory memory. Central olfactory tract conduction including the PC contains glutamatergic neurons and expresses TRPA1.^35^^,^^52^^,^^53^ Activation of TRPA1 or nicotine treatment has been reported to enhance MCL-1 expression via the ERK pathway, thereby exerting anti-apoptotic effects.^54^^,^^55^ The same mechanism may underlie our finding that nicotine inhibited apoptosis by stimulating TRPA1 in the central olfactory tract conduction in this study. TRPA1 KO mice showed similar tendency to WT mice in peripheral tract conduction. This suggests nicotine had little effect through TRPA1 in peripheral olfactory tract conduction, which possibly reflects the weak TRPA1 expression observed in the OE. In contrast, the improvement in olfactory memory by nicotine was abolished, while the detrimental effect of CSE was maintained in TRPA1 KO mice (Figures 2A and 6A). Taken together, the TRPA1-mediated nicotine effects were lost and overridden by CSE, leaving only adverse effects of CSE in central olfactory tract conduction of TRPA1 KO mice (Figure 7A).

Habituation is one form of olfactory memory.^56^ Nicotine treatment has been reported to suppress anxiety in response to social stimuli.^57^ In our habituation/dishabituation test, nicotine promoted habituation to conspecific mice in a TRPA1-dependent manner, supporting the notion that nicotine acts through TRPA1.

Olfactory memory tests before methimazole administration did not significantly differ between WT and TRPA1 KO control mice (*p* = 0.1688, Mann-Whitney U test) (Figures 2A and 6A). Furthermore, in histological examinations at the equivalent age of day 42 without any pharmacologic treatment, there was no significant difference in the number of apoptotic cells in the PC between WT and TRPA1 KO mice (Figure S4). TRPA1 deficiency is thus suggested to have little influence on the development of central and peripheral tract conduction. The finding that pharmacological inhibition of TRPA1 abolished the nicotine effect, similar to genetic KO (Figures 2A and 2B), further supports this assumption. Compared with age-matched groups without methimazole treatment, WT mice exhibited a higher number of apoptotic cells in the methimazole-exposed PBS group (*p* = 0.0417, Mann-Whitney U test) (Figures 3A and S4). Under conditions of neural olfactory impairment, nicotine treatment prominently suppressed TRPA1-dependent apoptosis in the PC and it improved olfactory memory (Figure 7B).

The role of TRPA1 in the central nervous system is divergently reported. TRPA1 activation was reported to induce neurotoxicity,^58^ whereas in a vascular cognitive impairment model by chronic cerebral hypoperfusion, it reportedly promoted myelination and exerted neuroprotective effects.^59^ In another, zinc sulfate-induced OE damage reduced blood flow in the central olfactory tract conduction.^60^ Therefore, in our methimazole-induced OE damage model, TRPA1 may have exerted neuroprotective effects, as observed in the vascular cognitive impairment model. Additionally, on day 7, TRPA1 expression levels in brain tissue were not affected by methimazole, nicotine, or CSE treatment (Figure S5), which suggests that the effects of TRPA1 may depend on its activation rather than on changes in expression.

While nicotine has known risks such as addiction, cardiovascular effects, and potential neurotoxicity, the therapeutic use of nicotine replacement therapy has been extensively evaluated for safety in clinical settings when used within defined doses and treatment periods.^61^ The potential to improve olfactory memory and mitigate dementia risk may outweigh the manageable risks once therapeutic conditions are optimized for clinical application. Olfactory disorders are a risk factor for dementia,^62^ and our findings demonstrate that nicotine may serve as a therapeutic strategy to reduce dementia risk by improving olfactory memory.

In summary, oral nicotine treatment inhibited apoptosis and improved olfactory memory by stimulating TRPA1 in central olfactory tract conduction. This study provides new insight into the effects of a cigarette-derived component on the nervous system.

### Limitations of the study

This study has several limitations. First, TRPA1 is expressed in multiple organs throughout the body, and the observed effects of nicotine on TRPA1 may not exclusively reflect olfactory tract conduction. Conditional KO mice confined to TRPA1 in olfactory tract conduction should be used in future studies. Second, as the statistical analyses relied on non-parametric tests, we could not rigorously evaluate genotype-by-treatment interactions. Future studies with larger sample sizes may enable a more robust assessment of the interactions. Third, this study evaluated TRPA1 function mainly at the protein level, so additional RNA-level analyses could further confirm the absence of changes in *Trpa1* gene expression.

## Resource availability

### Lead contact

Requests for further information and resources should be directed to and will be fulfilled by the lead contact, Muneki Hotomi (mhotomi@wakayama-med.ac.jp).

### Materials availability

This study did not generate new unique reagents.

### Data and code availability

• All behavioral and histological quantification data have been deposited at Figshare: https://doi.org/10.6084/m9.figshare.30673430 and are publicly available.
• This paper does not report original code.
• No additional resources were generated in this study.

## Acknowledgments

We acknowledge proofreading and editing by Benjamin Phillis, a Board-Certified Editor in the Life Sciences (BELS), at 10.13039/501100004420Wakayama Medical University. This work was supported by 10.13039/501100004330Smoking Research Foundation grant no. N285 (to M.H.), JSPS KAKENHI grant no. 25K02792 (to M.H.), JSPS KAKENHI grant no. 25K12748 (to H.S.), and JSPS KAKENHI grant no. 23K08992 (to M.K.).

## Author contributions

K.M., H.S., M.K., and M.H. developed the conception and design of the study; K.M. contributed to the acquisition of data; S.S. maintained and provided TRPA1 KO mice; K.M., H.S., and M.H. were responsible for the analysis and interpretation of data; H.S. wrote the manuscript with the help of A.A. and M.H.; and M.H. was responsible for revising the manuscript. All authors have contributed significantly to the work and approved the final version of the manuscript.

## Declaration of interests

The authors declare no competing interests.

## STAR★Methods

### Key resources table

**Table:** 

REAGENT or RESOURCE	SOURCE	IDENTIFIER
**Antibodies**		

Goat polyclonal anti-olfactory marker protein	FUJIFILM Wako	Cat#544-10001-WAKO; RRID: AB_664696
TRPA1 Polyclonal Antibody	Thermo Fisher Scientific	Cat# OST00061W-100U; RRID:AB_2209939

**Chemicals, peptides, and recombinant proteins**		

Methimazole	FUJIFILM Wako	CAS:60-56-0
Nicotine	Sigma-Aldrich	Cat#SML1236; CAS: 65-31-6
*In Situ* Cell Death Detection Kit, POD	Sigma-Aldrich	Cat#11684817910
Cigarette smoke extract	CMIC Pharma Science Co.	N/A
T&T Olfactometer	Daiichi Yakuhin Sangyo Co.	N/A
Decalcifying Solution B	FUJIFILM Wako	Cat#041-22031
HC-030031	FUJIFILM Wako	Cat#082-09963
Mouse Transient Receptor Potential Cation Channel Subfamily A Member 1 (TRPA1) ELISA Kit	Abbexa Ltd	Cat#abx551394
2,4,5-trimethylthiazole	Tokyo Chemical Industry Co., Ltd.	CAS:13623-11-5

**Deposited data**		

Raw and analyzed data	This paper	Figshare: https//doi.org/10.6084/m9.figshare.30673430

**Experimental models: Organisms/strains**		

Mouse: C57BL/6J	Charles River Laboratories Japan.	RRID:IMSR_JAX:000664
Mouse: B6;129P-Trpa1^tm1Kykw^/J	Jackson Laboratory.	RRID:IMSR_JAX:006401

**Software and algorithms**		

GraphPad Prism 10.0	GraphPad Software Inc.	RRID:SCR_002798

### Experimental model and study participant details

#### Ethical statement

This research adhered to the guidelines set by the National Science Foundation Animal Welfare Requirements and the Public Health Service Policy on the Humane Care and Use of Laboratory Animals. The experimental procedures and the use of mice were approved by both the Recombinant DNA Experiment Committee and the Animal Care and Use Committee at Wakayama Medical University (approval numbers: 1102 for the protocols and 2021-66 for the use of knockout mice).

#### Mice

Six-week-old male mice were used in this experiment to exclude potential effects of the estrous cycle on olfactory function and all were reared at the Wakayama Medical University Animal Experimental Facility. Wild-type C57BL/6J mice were purchased from Charles River Laboratories Japan, Inc. (Yokohama, Japan). TRPA1 KO mice (B6;129P-Trpa1^tm1Kykw^/J) of the same strain were purchased from the Jackson Laboratory (Bar Harbor, ME). TRPA1 KO mice are whole-body knockout mice (-/-) with C57/BL6 genetic background. The *Trpa1* gene was deleted using homologous recombination in embryonic stem cells.^63^

#### Experimental schedule

Methimazole (FUJIFILM Wako Pure Chemicals Co., Ltd., Osaka, Japan) dissolved in saline was administered to 6-week-old mice to induce OE damage. We used 75 mg/kg methimazole so that the ORN damage would be reversible.^40^^,^^64^ Day 0 was defined as the day of methimazole administration. Nicotine (Sigma-Aldrich Co., St Louis, MO) and CSE (CMIC Pharma Science Co., Ltd, Yamanashi, Japan) were used to investigate the effects of nicotine in tobacco products. WT mice and TRPA1 KO mice without methimazole treatment were defined as control mice. A separate cohort of WT and TRPA1 KO mice was treated with methimazole and subsequently divided into three groups: the PBS group, the CSE group, and the nicotine group. Each group received the agents on a daily basis from day 0 to day 42. The PBS group received 100 μL PBS intraorally, the CSE group received 1 μg/g CSE intranasally^65^ and the nicotine group received 2 μg/g nicotine dissolved in 100 μL PBS intraorally. Intraoral administration was performed by slowly delivering the solution into the mouse using a micropipette, allowing it to swallow voluntarily. Olfactory behavior tests were performed in 6-week-old control mice, and on days 7, 14, 28, and 42 (buried food test and olfactory memory test) and on day 7 (olfactory habituation/dishabituation test) in the three treatment groups (PBS, CSE, and nicotine). In addition, a subgroup of the nicotine group received the 100 μg/g TRPA1 antagonist HC-030031 intraperitoneally daily from day 0 to day 7,^66^ and underwent the olfactory memory test and olfactory habituation/dishabituation test on day 7. This group was designated as the nicotine + TRPA1 inhibitor subgroup. Histological examinations were performed on day 42. To assess the effects of systemically administered nicotine, WT mice were further assigned to the PBS i.p. group or the nicotine i.p. group. These groups received intraperitoneal injections of 100 μL PBS or 2 μg/g nicotine, respectively, once daily from day 0 to day 42. Olfactory memory tests were performed on days 7, 14, 28, and 42.

### Method details

#### Olfactory behavioral testing

In this study, we employed the following behavioral tests: buried food test, olfactory memory test, olfactory habituation/dishabituation test, and avoidance behavioral test. All test cages and boxes were placed in the same location within the same room. For each test, identical types of new equipment were used to maintain consistency. During testing, the environment was kept quiet and access by others was restricted. All tests were conducted by the same examiner to minimize environmental confounding factors.

#### Buried food test

Buried food test was conducted to assess odor detection.^67^ Briefly, mice were fasted for 48 h before the test was carried out. Each set of tests was conducted twice, 24 and 48 h after the start of fasting. Approximately 10 min before the start of each test, the mice were moved to a new cage where one food pellet had been placed. This initiation process reminds the mice of the food smell. The size of test cages was 38 × 24 × 20 cm and they were filled with 5 cm thick bedding. Food pellets were buried at a depth of 1 cm in one of the four corners. After initiation, the mice were placed in the center of the test cage and the test began. Latency was defined as the time required for the mouse to find the food pellet. If a mouse could not find the pellet within 300 s, the latency was recorded as 300 s.

#### Olfactory memory test

Olfactory memory test reflects the function of olfactory memory, which involves central olfactory tract conduction. In this study, the test was conducted based on previous reports with slight modifications.^12^^,^^68^ The 72 × 60 × 30 cm test box had small boxes (11 × 8 × 2 cm) in each of the four corners. These small boxes were filled with the following differently scented beddings: β-phenylethyl alcohol, methylcyclopentenolone, isovaleric acid, and γ-undecalactone from the T&T Olfactometer.^69^ One of the four small boxes was then randomly selected and a piece of chocolate was hidden under the scented bedding. Mice were transferred to a new cage 10 min before the test and given a piece of chocolate to remind them of the chocolate smell. On Day 1, mice were released into the center of the test cage and the time until they found the chocolate was recorded, up to a limit of 300 s. The same test was carried out again the following day (Day 2) in which the chocolate was then hidden in a small box with the same smell as on the previous day. The ratio of the time of Day 2 to Day 1 (Day 2/Day 1 Ratio) was recorded.

#### Olfactory habituation/dishabituation test

Olfactory habituation/dishabituation test is a behavioral test for odor discrimination that reflects central olfactory tract conduction.^38^^,^^70^ This test was conducted with reference to previous reports.^71^ The mice were transferred to a cage measuring 32×24×10 cm and were allowed to acclimate for 30 min. A cotton swab of 16 mm was prepared as an applicator for odor presentation, and a clean applicator was inserted during the acclimation period to reduce exploratory behavior caused by novelty. After the acclimation period, the applicator was used to present odor stimuli, including water and a cage wipe extract from conspecifics. Water was used as a control and the cage wipe extract was used as social odor. Specifically, each odor stimulus was presented three times in the aforementioned order, with each presentation lasting 2 min. A 1-min interval was set between each presentation. The time spent sniffing each odor stimulus was recorded during the stimulation period.

#### Avoidance behavioral test

Mice were sequentially transferred to new cages at 30 min intervals, and this process was repeated three additional times to acclimate them to a novel environment. For the avoidance test, a test cage was placed in a ventilated area, and filter paper containing 4 μL of 2,4,5-trimethylthiazole (nTMT; Tokyo Chemical Industry, Tokyo, Japan) was affixed to one side of the cage. The cage was then sealed with a lid to minimize odor diffusion. At the start of the test, each mouse was introduced into the cage, and its freezing behavior, defined as complete immobility lasting more than 3 seconds, was recorded for a total duration of 5 min.^72^

#### Preparation of tissue sections

Whole skulls of euthanized mice were obtained to prepare nasal and brain tissue sections. The specimens were fixed using 4% paraformaldehyde and then decalcified using Decalcifying Solution B (FUJIFILM Wako, Japan). Nasal coronal sections containing the center of the eye were used to assess OE, ORNs and OB. A brain coronal section was taken 2 mm ventral to the bregma for assessment of the PC. Each slice was cut from a paraffin block at a thickness of 5 μm. These sections were then used for hematoxylin-eosin staining or immunostaining.

#### Immunohistochemical staining

In this study, we used the following primary antibodies, diluted in PBS: anti-olfactory marker protein (OMP; goat polyclonal, 1:8000 dilution; FUJIFILM Wako) and anti-TRPA1 polyclonal antibody (rabbit polyclonal,1:250 dilution; Thermo Fisher Scientific, Waltham, MA). Immunohistochemical staining was outsourced to Morpho Technology (Hokkaido, Japan). TUNEL staining was conducted using the *In Situ* Cell Death Detection Kit, POD (Sigma-Aldrich Co., St. Louis, MO), following the manufacturer’s instructions.

#### Histological analysis

The nasal septum was used to measure OE thickness and the positive rate of OMP-positive cells. In accordance with previous reports, the thickness of the right and left nasal septal mucosa was measured at 250, 500 and 750 μm from the upper edge of the nasal septum in each mouse. In other words, OE thickness was measured at six locations per mouse.^64^^,^^68^ For the OMP-positive cell rate, the ratio of OMP-positive cells/total cells was counted within 100 μm of OE on the nasal septum.^40^ Coronal sections containing PC were used to assess apoptosis in the central olfactory tract conduction. The number of TUNEL stain-positive cells was counted within the whole coronal sections. Superficial cells along the pia mater were excluded from the counts.

#### Enzyme-linked Immunosorbent Assay

The brain tissue was collected on day 7 from WT mice in the PBS, CSE, and nicotine groups, as well as a 7-week-old untreated group. The untreated group received no pharmacological treatment, including methimazole. The age of the untreated group corresponded to day 7 in the other three groups. Each sample tissue was homogenized in 1 mL PBS and centrifuged at 500 × g for 5 minutes to obtain 200 μL of supernatants. The concentrations of TRPA1 were measured in pg/mL based on standard curves using ELISA kits (Proteintech, Rosemont, IL) according to the manufacturer’s instructions.

### Quantification and statistical analysis

#### Statistical analyses

All statistical analyses were carried out using GraphPad Prism 10.0 (GraphPad Software Inc., San Diego, CA). Normality and variance equality were assessed using the Shapiro-Wilk test and Levene’s test, respectively. No datasets satisfied the assumptions of normality, so non-parametric tests were employed. Differences were therefore determined by Mann-Whitney U test or Fisher’s exact test (comparison of two groups) or Kruskal-Wallis test with Dunn’s post hoc analysis (comparison of multiple groups). *P*<0.05 was considered to be statistically significant.
