# Supplementary material for: The impact of nicotine on the olfactory memory and its relationship with TRPA1
Source: iScience. 2026 Feb 7;29(3):114908. doi: 10.1016/j.isci.2026.114908 (PMC12936849; doi:10.1016/j.isci.2026.114908)
Supplement: Document S1. Figures S1–S5 [file mmc1.pdf]

iScience, Volume *29*

Supplemental information

The impact of nicotine on the olfactory memory

and its relationship with TRPA1

Kazuya Mizobata, Hideki Sakatani, Masamitsu Kono, Shizuya Saika, Akinori Akaike, and Muneki Hotomi

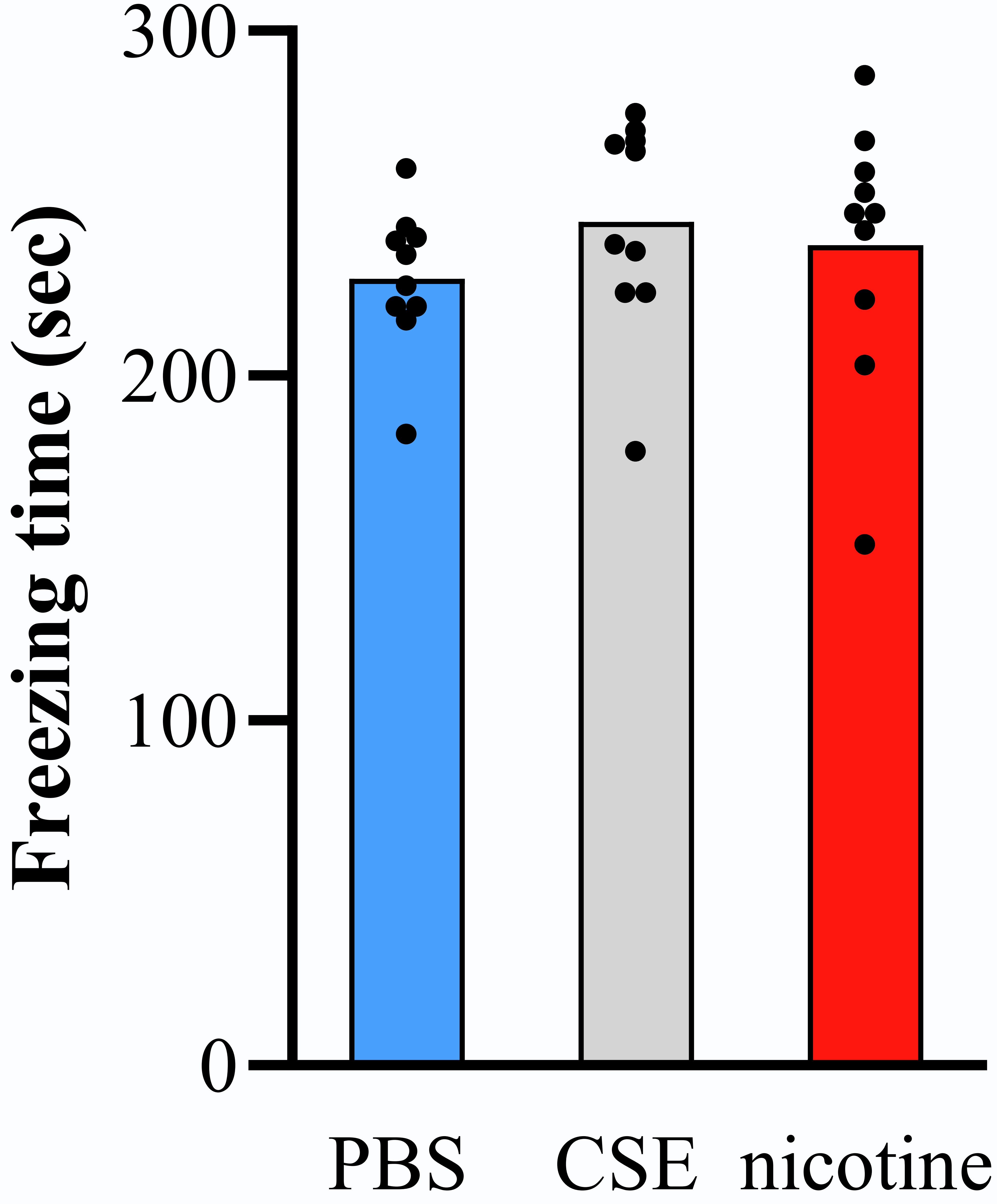

Figure S1. Avoidance behavioral test, related to Figures 2A and 2B

To assess anxiety levels as a potential confounding factor in olfactory behavioral testing, the avoidance behavioral test was conducted in WT mice on day 7. A longer freezing time indicates a higher level of anxiety. One dot represents one mouse. PBS group (n=10), CSE group (n=10), nicotine group (n=10). Statistical comparisons to PBS group were performed using Kruskal-Wallis test with Dunn's multiple-comparison test.

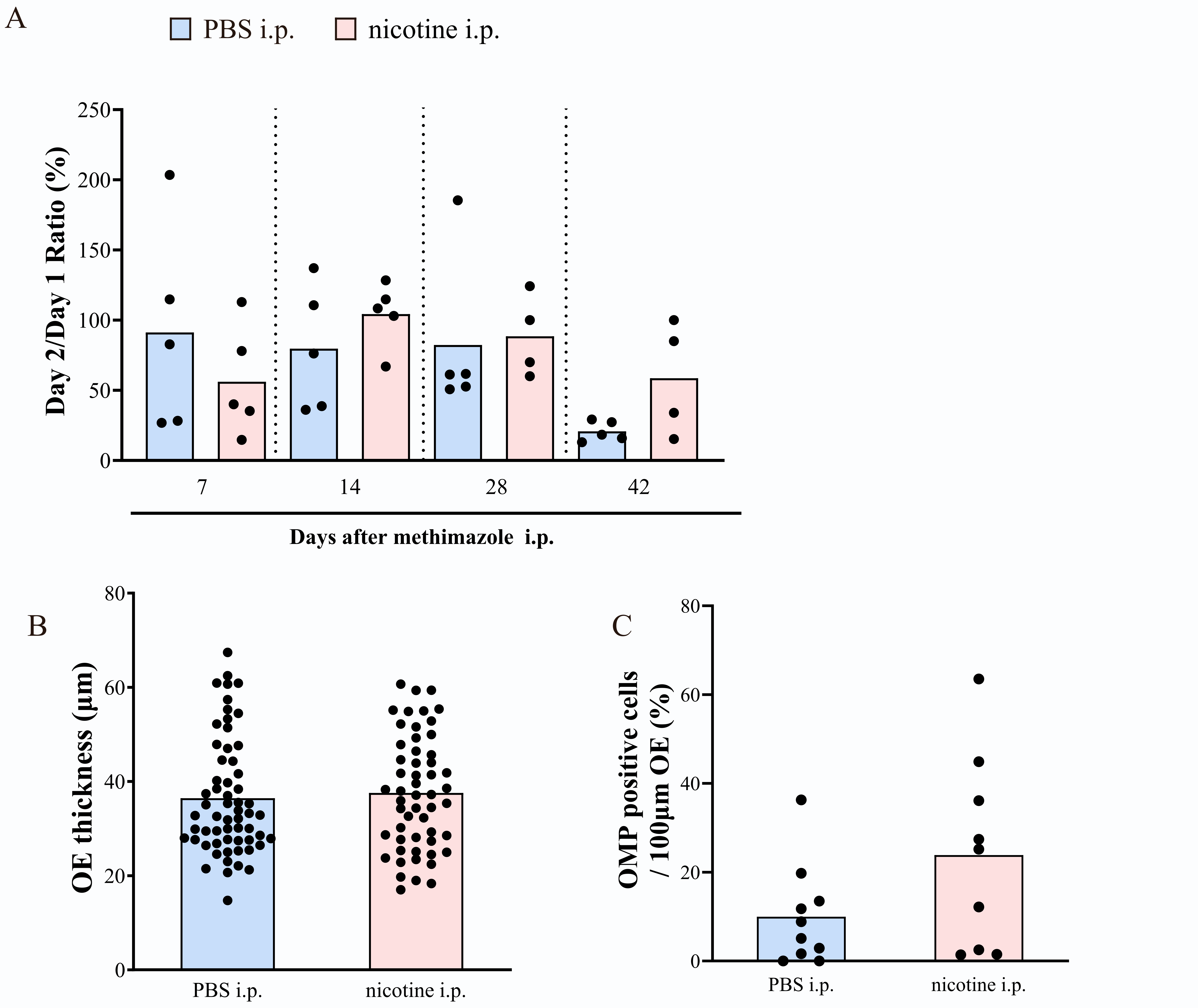

Figure S2. Systemic nicotine administration did not affect olfactory tract conduction in wild-type mice, related to Figures 2
The effects of nicotine administered intraperitoneally on peripheral and central olfactory tract conduction in WT mice. (**A**) Olfactory memory test. The ratio of two consecutive days per mouse. One dot represents one mouse. PBS i.p. group (n=5), nicotine i.p. group (n=4-5). (**B**) OE thickness on day 42. Six dots per mouse are plotted. PBS i.p. group (n=10), nicotine i.p. group (n=9). (**C**) OMP-positive cell rate on day 42. PBS i.p. group (n=10), nicotine i.p. group (n=9). Statistical comparisons were performed using Mann–Whitney U test.

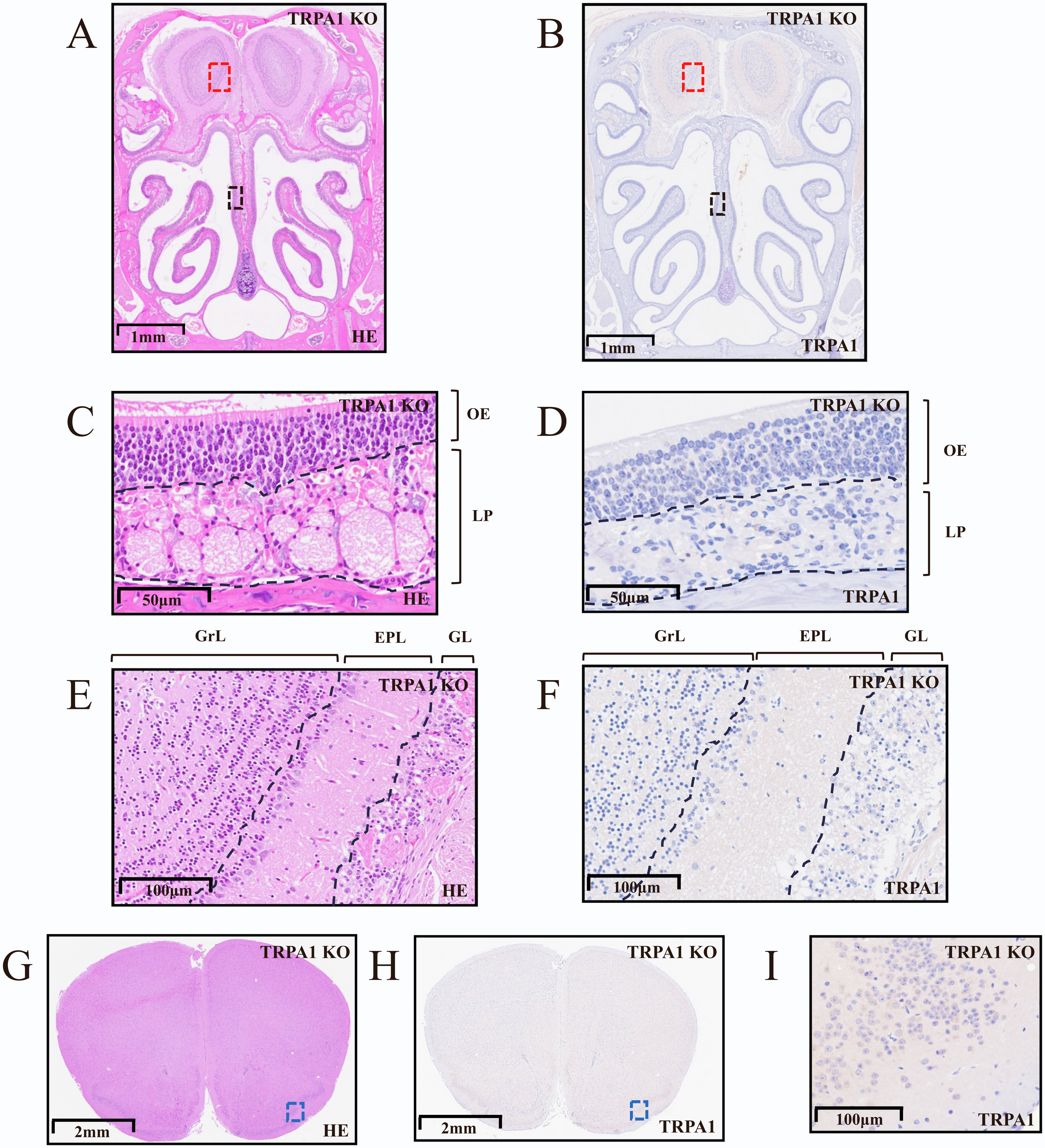

Figure S3. Validation of TRPA1 antibody staining in TRPA1 KO mice, related to Figures 4

Representative histological images of TRPA1 KO mice before methimazole administration, using the same method as in Figure 4. (**A-F**) Coronal sections of nasal cavity. (**G-I**) Coronal sections of brain including PC. (**A, C, E, G**) HE-stained images. (**B, D, F, H, I**) anti-TRPA1 antibody-stained images. (**A, B**) Low magnification images of nasal cavity. The area surrounded by the black and red dashed lines represent the OE and the OB, respectively. (**C, D**) Highly magnified images of OE in the black boxes in **A** and **B**. (**E, F**) Highly magnified images of the OB in the red boxes of **A** and **B**. (**G, H**) Low magnification images of brain. The area enclosed by the blue dashed line represents one part of PC. (**I**) Highly magnified image of PC in the blue box of **H**. Scale bars represent (**A, B**) 1 mm, (**C, D**) 50 µm, (**E, F**) 100 µm, (**G, H**) 2 mm and (**I**) 100 µm respectively. TRPA1: Transient receptor potential ankyrin 1; HE: hematoxylin-eosin; OE: olfactory epithelium; OB: olfactory bulb; LP: lamina propria; GrL: granule cell layer; EPL: external plexiform layer; GL: glomerular layer; PC: piriform cortex.

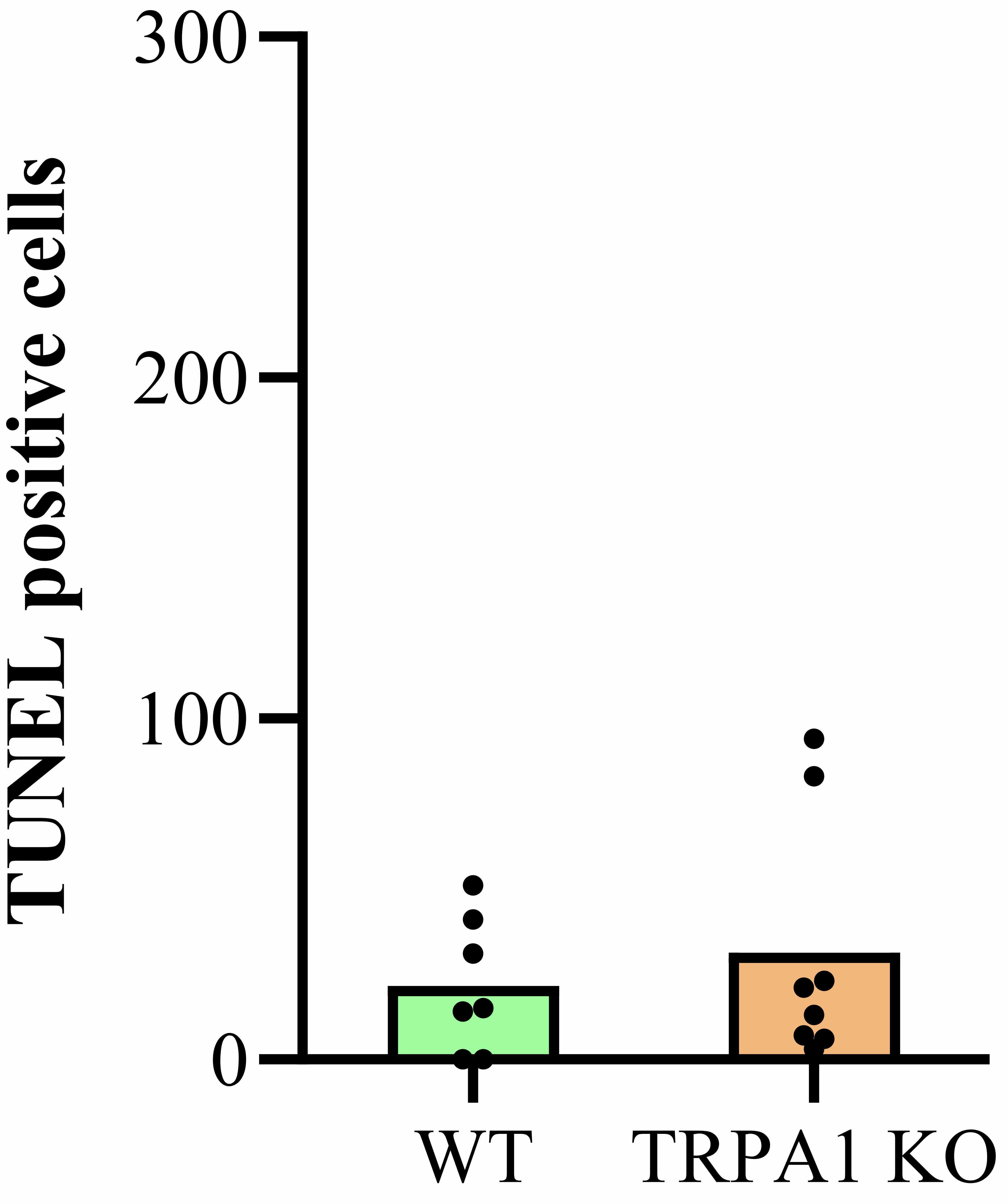

Figure S4. Histological comparison between WT and TRPA1 KO mice without methimazole treatment, related to Figures 3A and 6C

13-week-old WT and TRPA1 KO mice without any pharmacological treatment (methimazole, PBS, CSE, or nicotine) were sacrificed. Brain tissues were collected and TUNEL-positive cells in the PC were quantified as described in Figures 3A and 6C. 13 weeks of age corresponds to day 42 in the PBS, CSE, and nicotine groups. WT mice (n=7), TRPA1 KO mice (n=8). Statistical comparisons were performed using the Mann–Whitney U test.

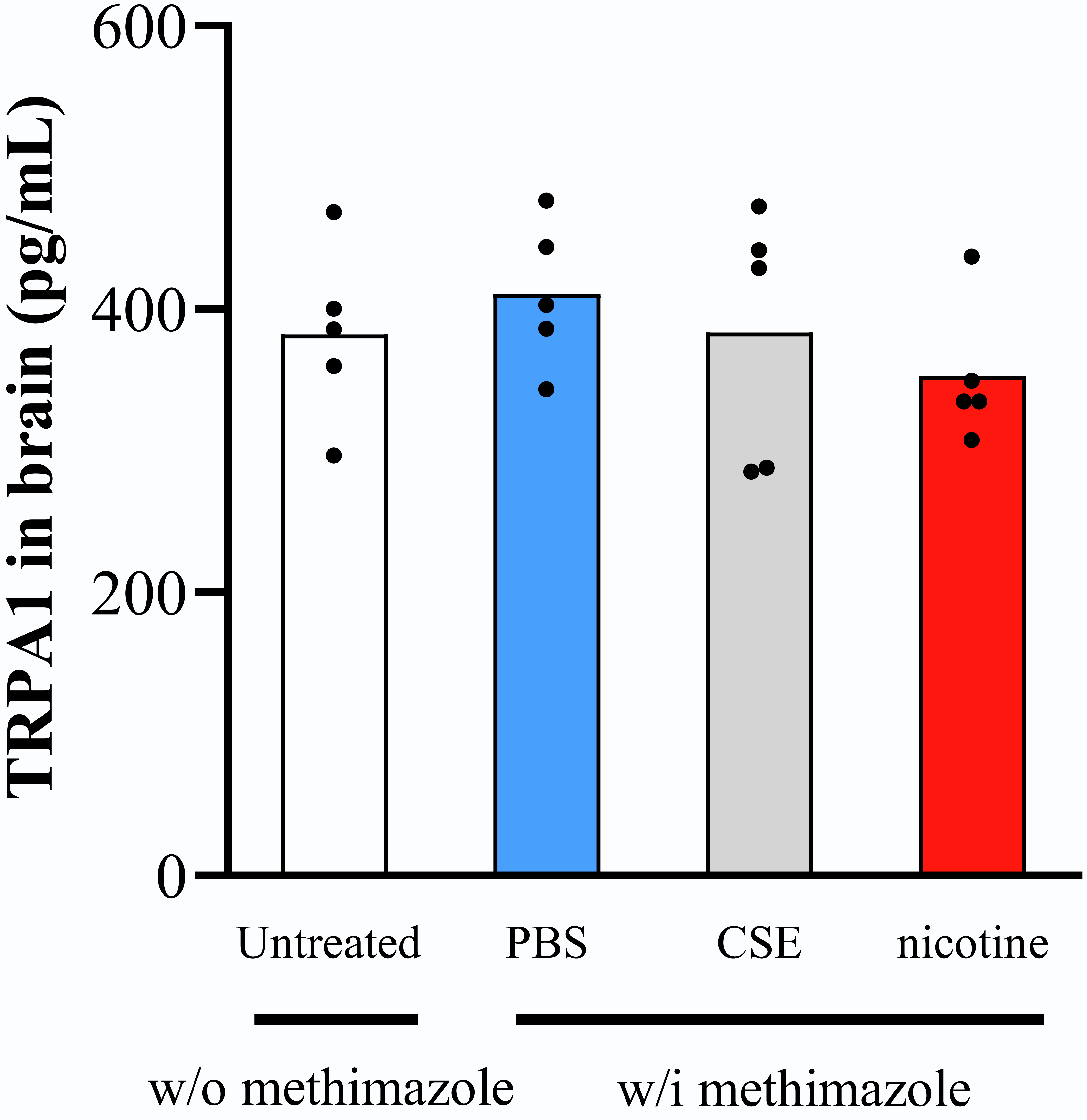

Figure S5. TRPA1 expression levels in brain tissue measured by Enzyme-Linked Immunosorbent Assay, related to Figures 2

TRPA1 expression levels in brain tissue were measured by Enzyme-Linked Immunosorbent Assay (ELISA) in the PBS, CSE, and nicotine groups of WT mice on day 7. An untreated group of 7-week-old mice without methimazole (corresponding to day 7 of the three groups) was included as a control. One dot represents one mouse. Untreated group (n=5), PBS group (n=5), CSE group (n=5), nicotine group (n=5). Statistical comparisons with the untreated group were performed using the Kruskal-Wallis test followed by Dunn's multiple-comparison test.
